# The rs16906252:C>T SNP is not associated with increased overall survival or temozolomide response in a Han-Chinese glioma cohort

**DOI:** 10.1371/journal.pone.0178842

**Published:** 2017-06-02

**Authors:** Kuo-Chen Wei, Chia-Yuan Chen, Li-Ying Feng, Wei-Tzu Huang, Chia-Hua Chen, Peng-Wei Hsu, Kai Wang, Leroy E. Hood, Leslie Y. Chen

**Affiliations:** 1 Department of Neurosurgery, Chang Gung Memorial Hospital-Linkou Medical Center, Taoyuan, Taiwan, Republic of China; 2 College of Medicine, Chang Gung University, Taoyuan, Taiwan, Republic of China; 3 Department of Medical Research and Development, Chang Gung Memorial Hospital-Linkou Medical Center, Taoyuan, Taiwan, Republic of China; 4 Institute for Systems Biology, Seattle, Washington, United States of America; University of Navarra, SPAIN

## Abstract

The methylation status of O-6-methylguanine-DNA methyltransferase (MGMT) is associated with the prognosis in gliomas and in other cancers. Recent studies showed that rs16906252, an SNP in the *MGMT* promoter, is associated with promoter methylation and is a predictor of the overall survival time (OST) and the response to temozolomide (TMZ) treatment. However, these findings haven’t been systematically investigated in the Han-Chinese population. We analyzed the relevance between rs16906252 polymorphisms, the *MGMT* methylation status, and the OST in 72 Han-Chinese gliomas patients. The *MGMT* promoter methylation was measured by bisulfite conversion followed by pyro-sequencing, while rs16906252 was measured by restriction endonuclease digestion. Contrary to the previous findings, we found no association between rs16906252 genotypes and promoter methylation on *MGMT*. The lower-grade glioma (LGGs) patients carrying the C allele with rs16906252 showed a surprisingly better OST (P = 0.04). Furthermore, the LGG patients carrying hypo-methylated *MGMT* promoter and rs16906252 T allele showed significantly poorer prognosis. The prognostic benefit of *MGMT* promoter methylation and genotypes on gliomas patients is marginal. A new molecular stratified patient grouping of LGGs is potentially associated with poorer OST. Active MGMT might have a protective role in LGG tumors, enabling evolution to severe malignancy.

## Introduction

The annual incidence of gliomas slowly increases and is approximately 5 cases per 100,000 individuals [1]. In humans, gliomas include the glioblastomas and several other types of lower-grade gliomas (LGGs). More than 80% of malignant gliomas belong to glioblastomas, with an average survival time of 14 months [2]. The standard treatment of glioblastomas includes maximal surgical resection, and radio- and chemotherapy. Frequently used anti-tumor drugs to treat glioblastomas include Temozolomide (TMZ) and Bevacizumab [3, 4]. The LGGs accounts for 15% of total primary brain tumors and approximately 2,000 to 3,000 LGGs are diagnosed in the United States every year [5, 6].

Promoter methylation of O-6-methylguanine-DNA methyltransferase (MGMT) is an important clinical predictor to overall survival time (OST) in gliomas and other cancers including colorectal cancer, lymphoma and lung cancer [7–10]. MGMT protein abundance is a key indicator of the TMZ treatment response and is routinely assessed by measuring promoter methylation of *MGMT* [11–13]. Recent studies suggested the rs16906252 genotype in tumors is associated with *MGMT* promoter methylation [8, 14, 15]. Compared to the wild type of homozygous C, glioblastomas that carry the T allele of rs16906252, i.e. heterozygous C and T or homozygous T alleles, showed hyper-methylated *MGMT* promoters and a better survival outcome [16].

The studies supporting the clinical association on the tumoral rs16906252 polymorphisms and promoter methylation of *MGMT* are primarily on white patients. Interestingly, the T allele of the rs16906252 is 6.857% in whites according to the Exome Aggregation Consortium (ExAC) but is not seen in East Asia [17]. This study aims to investigate the rs16906252 polymorphisms in Han-Chinese and evaluate the clinical relevance of the rs16906252 genotype, the *MGMT* promoter methylation in the brain cancer patients.

## Materials and methods

### Sample collection

The study was approved by the institutional review board (IRB) of the Chang Gung Memorial Hospital (CGMH). All patients were provided with written informed consent. Seventy-two patients were recruited between 2005 and 2015 with pathologically confirmed malignant gliomas including glioblastomas (WHO grade IV) and LGGs (WHO grade II and III). Tumor tissues were surgically resected, snap frozen in liquid nitrogen, and stored at -80°C. Matched control samples were collected from peripheral blood monocytes (PBMCs) during surgery.

### Genomic DNA extraction

Tumor DNA were extracted by using the Gentra Puregene kit (QIAGEN) following the manufacturer’s protocol. In short, the tumor tissues were lysed with lysis solution using proteinase K treatment. After protein precipitation by protein precipitation solution and DNA precipitation by isopropanol, DNAs were washed with 70% ethanol. The pellet was air-dried and re-suspended in Tris-EDTA buffer or deionized water. Blood DNA extraction began with separation and lysis of lymphocytes from whole blood by using a hypotonic buffer. After PBS wash, lymphocytes were treated with lysis solution and proteinase K. DNA were extracted by phenol and chloroform separation. The same procedure for DNA precipitation and re-suspension was followed as that of tumor DNA.

### SNP genotyping

The *MGMT* promoter region was amplified by polymerase chain reaction (PCR) with forward and reverse primer pairs 5’-GCCCCTAGAACGCTTTGCGTC-3’ and 5’-AGACACTCACCAAGTCGCAACG-3’ at 65^°^C of annealing temperature in a master mix enzyme (New England Biolabs). Expected length of the PCR amplicons is 74 base pairs (bps) with or without a Hha1 site depending on the rs16906252 genotypes. The PCR amplicons were treated with the *Hha*1 enzyme (New England Biolabs), which recognizes the CGCG site presented with the C allele of rs16906252. The result of Hha1 treatment was then analyzed by electrophoresis on 3% agarose gel. PCR amplicons with a T variant remained uncut full length (74 bps), while those with the C allele were fragmented into 48 bps and 26 bps.

### Bisulfite conversion and pyrosequencing

To determine promoter methylation, 500 ng tumor DNA were biosulfite-converted, amplified by the PyroMark MGMT kit (Qiagen) followed by pyrosequencing analysis. Five potentially methylated sites in the first exon (+17 to +39) of the *MGMT* gene were analyzed by the EZ DNA Methylation-Gold^TM^ Kit (Zymo Research) following the manufacturer’s suggestions. The methylation level was analyzed on the PSQ96 MA system (Qiagen) and quantified by the Qiagen Pyro-Mark CpG software (Qiagen). Hyper-methylation is defined by the average methylation sites equal or higher than 10% on the 5 CpG, otherwise regarded as hypo-methylation.

### Statistical analysis

Statistical analyses were performed by the GraphPad prism 5 software. Overall survival time (OST) was defined as the period from the date of tumor removal to the date of death and was analyzed by the Kaplan–Meier and Log-rank test for statistical significance. Chi-square analysis was applied to test the association between the methylation status and the rs16906252 genotypes. *P*-values of lower than 0.05 were considered significant.

## Results

### Clinical characteristic of the glioma cohort

Seventy-two glioma patients, including 34 glioblastomas and 38 LGGs, between 2005–2015, were recruited in this study including 44 newly diagnosed and 28 relapsed patients. The mean age of all glioma patients was 48.9 years old (22–95 years old) and 59.7% was male. Sixteen patients received gross total resection, whereas the others received nearly total, subtotal, or partial removal of tumors (77.8%). Nearly half of the patients (41.7%), mostly the glioblastoma patients received the TMZ chemotherapy. Forty-four patients passed away with recorded OSTs, whereas 28 patients were censored due to either being alive at the time of analysis or loss of contact. Table 1 summarizes the clinical statistics of the patient cohort. The *MGMT* promoter methylation analysis suggested that slightly more than half (51.4%) of the patients showed hyper-methylated *MGMT* promoter. Regarding *MGMT* genotyping, 29.2% tumors (21 of 72) carried the T allele at rs16906252, while only 18.2% (12 of 66) carried the same allele in control DNA. The allele frequency of the rs16906252 minor allele T in tumor samples was 15.3% and 9.8% in the matched-germline control samples. All relevant data of participant-level is summarized in S1 Table.

**Table 1:** Summary of the demographic and molecular characteristics of gliomas patients.

	Patients	%
Age*, **mean (range)**	48.9 (22–95)	
<50	39	54.2 %
≥50	33	45.8 %
**Sex**		
Male	43	59.7 %
Female	29	40.3 %
**Pathology**		
glioblastomas	34	47.2 %
LGGs	38	52.8 %
**Treatment**		
With TMZ	30	41.7 %
Without TMZ	42	58.3 %
**Treatment received**		
Gross resection	16	22.2 %
Others^#^	56	77.8 %
**At diagnosis**		
Newly diagnosed	44	61.1 %
Recurrent	28	38.9 %
**MGMT methylation**		
Hyper-methylation	37	51.4 %
Hypo-methylation	35	48.6 %
**Tumor rs16906252**		
CC	51	70.8 %
CT	20	27.8 %
TT	1	1.4%
**Germline rs16906252**		
CC	54	81.8 %
CT	11	16.7 %
TT	1	1.5%

Abbreviations: LGG, lower-grade gliomas.

*Age at diagnosis.

^#^Others include nearly total, subtotal, and partial resection.

### rs16906252 genotypes are not associated with promoter methylation in *MGMT*

**Fig 1:**
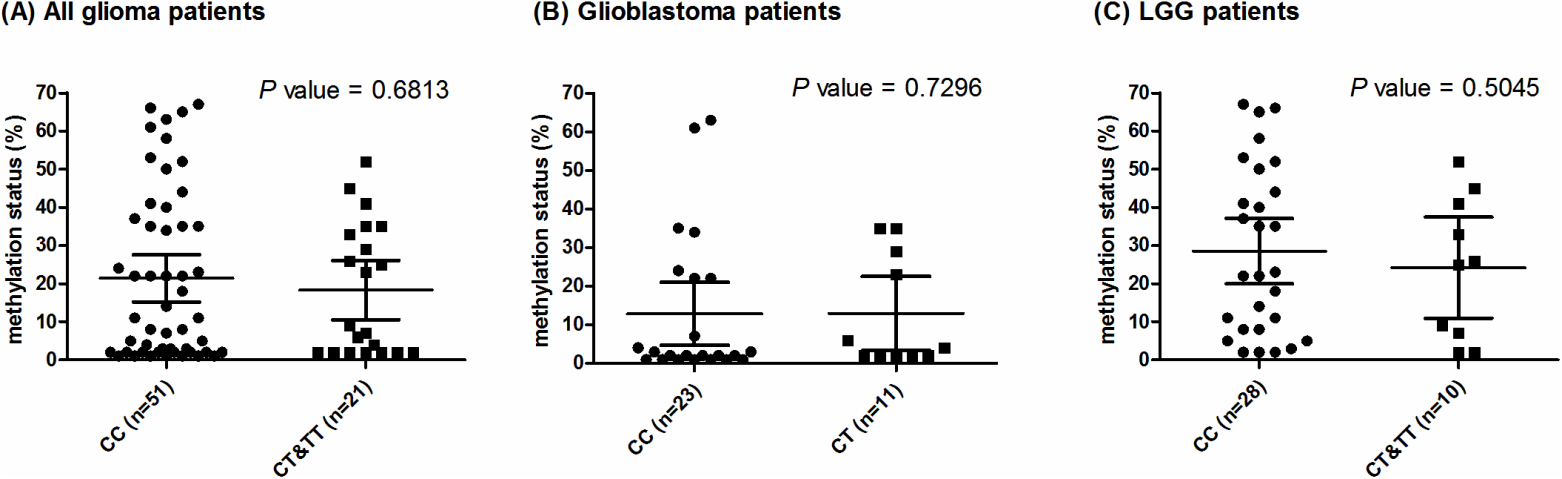
Correlation between the rs16906252 genotype and promoter methylation. *MGMT* promoter methylation and the rs16906252 genotype were analyzed for association with (A) all glioma patients (P = 0.5573), (B) glioblastoma patients (P = 0.9899), and (C) LGG patients (P = 0.5827). Neither all nor subgroups of patients replicated the previously reported association in the Han-Chinese.

Previous studies suggested that the rs16906252 T allele is associated with methylated promoter in *MGMT* in lung [7], colorectal [8, 18], and brain cancer [16]. However, the association is not replicated in our Han-Chinese gliomas cohort. The *MGMT* promoter showed an average methylation of 21.5% and 18.3% in the patients carrying the CC (N = 51) and CT and TT (N = 21) genotypes, respectively (Fig 1A). When the glioblastomas (Odds Ratio [OR]: 0.7656; 95%Confidence Interval [CI]: 0.5426–1.542; P = 0.7296, S2 Table) and LGG patients (OR: 1.667; 95%CI: 0.3689–7.529; P = 0.5045, S2 Table) were analyzed separately, none of the correlation was significant in either group of patients (Fig 1B and 1C and S2 Table).

### rs16906252 predicts OST on LGG patients

**Fig 2:**
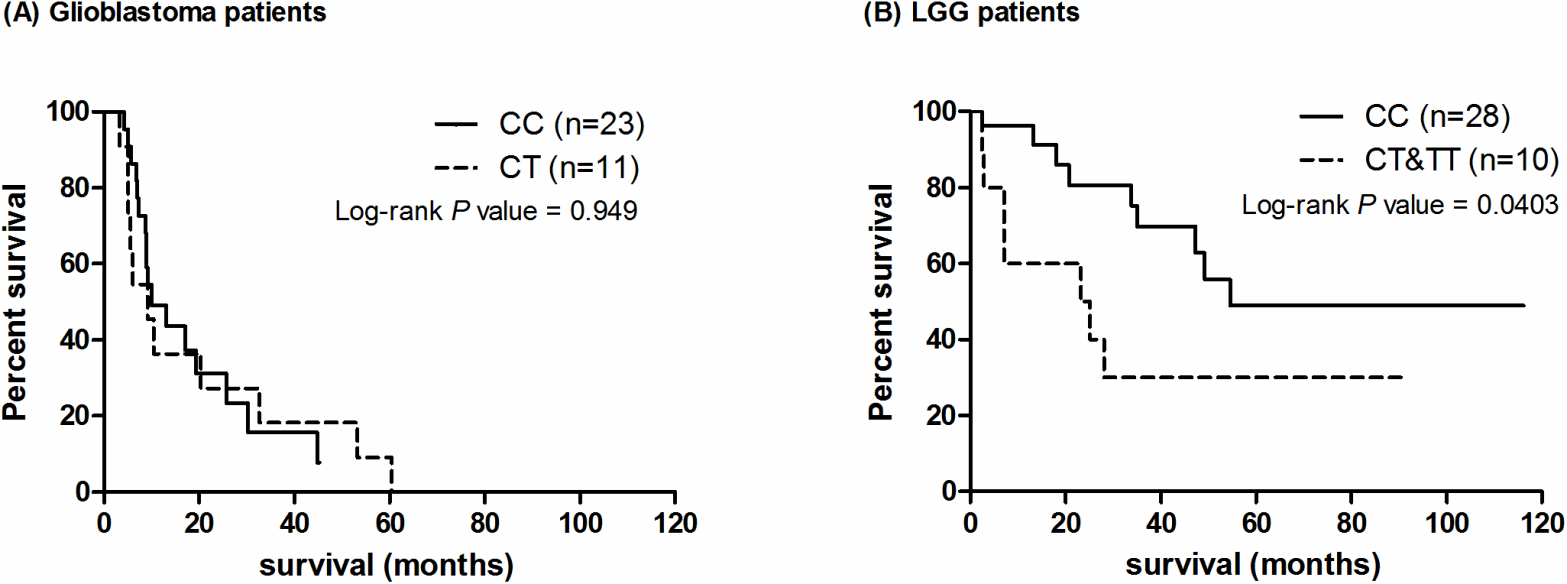
**Kaplan-Meier survival analysis of the rs16906252 genotype on (A) glioblastoma patients and (B) LGG patients.** (A) Previous studies suggested that the T allele is associated with a prolonged OST. However, the association is not observed in glioblastoma patients (P = 0.949). (B) LGG patients with the homozygous C alleles showed a significantly prolonged OST compared to that with CT or homozygous T alleles (P = 0.0403).

The rs16906252 genotypes in tumors are not correlated with OST in glioblastoma patients (HR: 0.9733; 95%CI: 0.6218–1.559; P = 0.949, S3 Table). The median survival time of patients with the CC and CT genotypes was 10 months (n = 23) and 9.2 months (n = 11), respectively, while none of the patients carried the TT genotype (Fig 2A). However, in LGG patients, the CC genotype is correlated with a longer OST (HR: 0.2902; 95%CI: 0.08898–0.9467; P = 0.0403, Fig 2B and S3 Table). Patients carrying the CC genotype had a longer median OST (54.6 months; n = 28) compared to those with the CT genotype (24.2 months; n = 10, p = 0.0403). Our results suggest that although not observed in glioblastoma patients, the CC genotype showed a contradictive longer OST in LGG patients [15].

### MGMT promoter methylation is not correlated to OST

**Fig 3:**
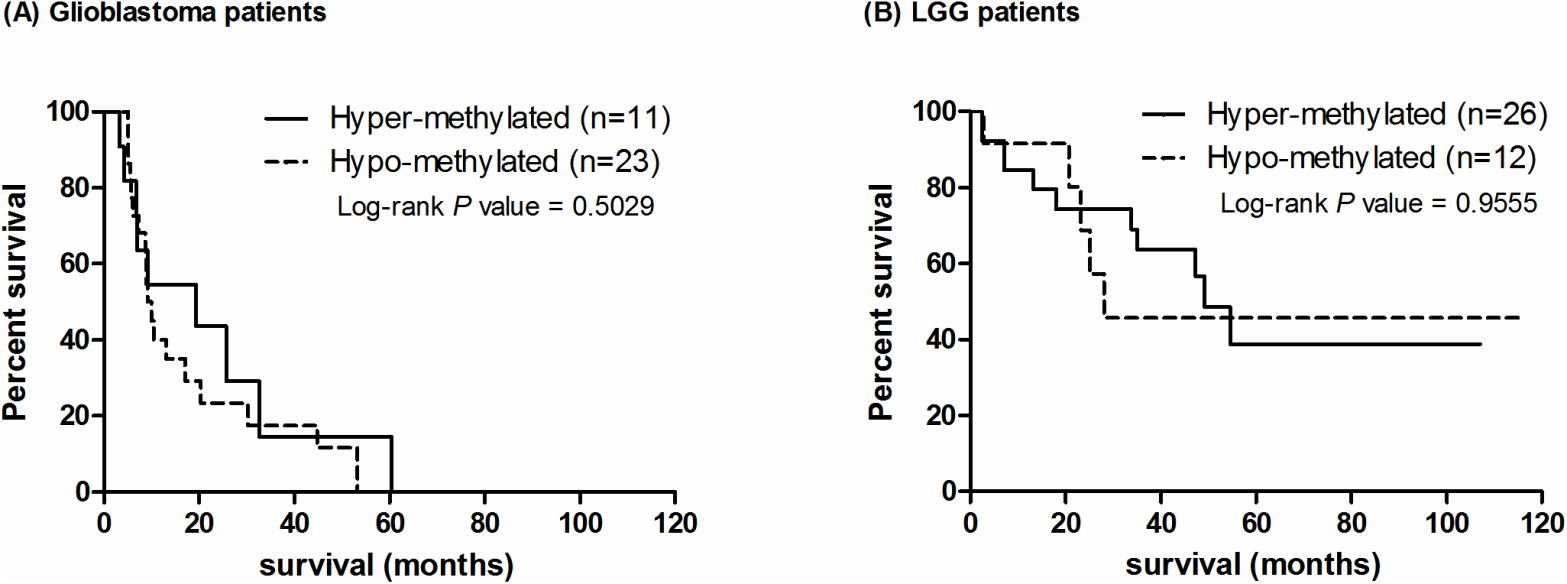
Kaplan-Meier survival analysis on the extent of *MGMT* promoter methylation. In glioblastoma patients, most patients (68%) showed hypo-methylation, but this was opposite in LGG patients (32%). Statistical analysis suggests that the OST is not associated with *MGMT* promoter methylation on (A) glioblastoma patients (P = 0.5029) and (B) LGG patients (P = 0.9555).

Although repeatedly reported as a good predictor, *MGMT* promoter methylation is not correlated with OST on both glioblastomas and LGG patients in our results (Fig 3). In the glioblastoma cohort, the median survival time of methylated and non-methylated patients was 19.3 months (n = 11) and 9.6 months (n = 23), respectively (HR: 0.7617; 95%CI: 0.3436–1.689; P = 0.5029, Fig 3A and S3 Table). In the LGGs cohort, the median survival time of methylated and non-methylated patients was 49.2 months (n = 26) and 28.1 months (n = 12), respectively (HR: 1.031; 95%CI: 0.3572–2.973; P = 0.9555, Fig 3B and S3 Table). Regardless of the methylation status, circa 40% LGG patients in each group were still alive at the time of analysis. Our results suggest that promoter methylation is not correlated with OST in Han-Chinese ethnicity.

### rs16906252 genotypes stratify LGG patients with hypo-methylated *MGMT*

**Fig 4:**
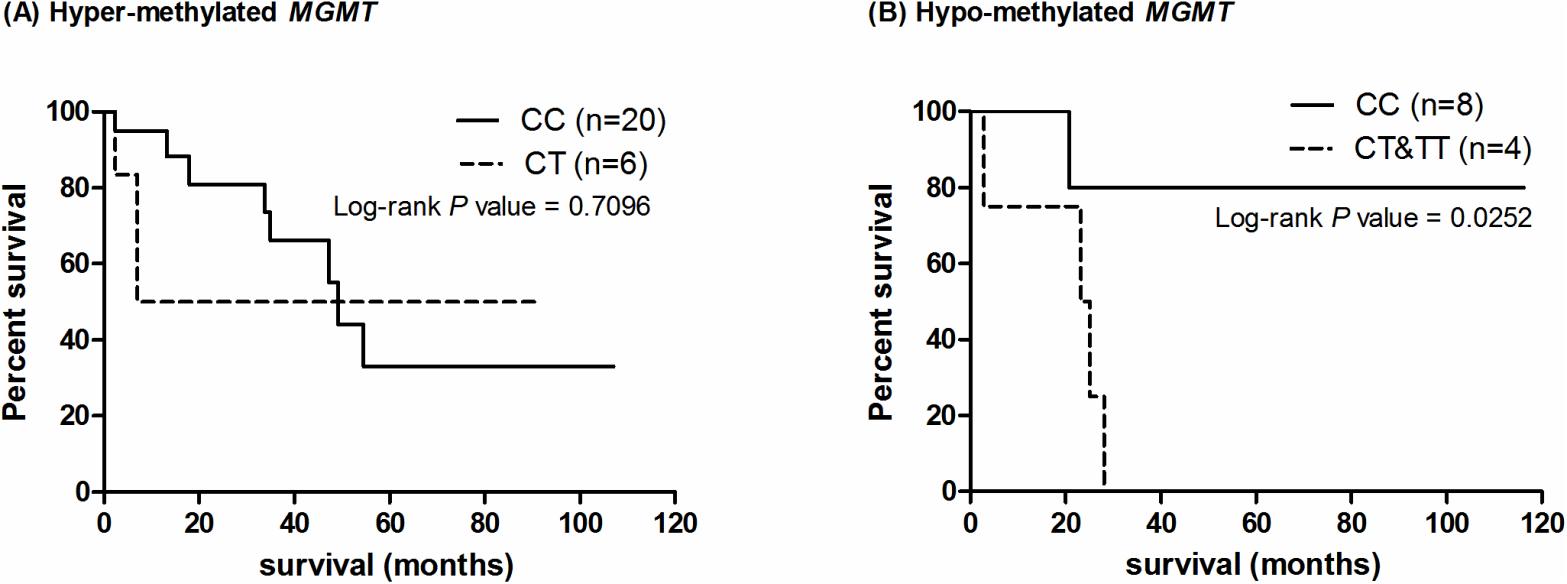
OST and the *MGMT* promoter genotype and methylation on LGGs. The LGG patients were further classified with (A) hyper- and (B) hypo-methylated promoter in *MGMT*. The C (or T) allele is not associated with a prolonged OST on patients with methylated promoter (A, P = 0.7096) but on patients of hypo-methylation (B, P = 0.0252).

We further tested the correlation between OST and the combination of methylation and genotypes with *MGMT*. The rs16906252 genotypes surprisingly stratify the LGG patients with hypo-methylated *MGMT* promoter (Fig 4). The patients with hyper-methylated *MGMT* and homozygous C genotype on rs16906252 demonstrated better OST compared with the heterozygous CT genotypes, but this association is not statistically significant (HR: 0.7628; 95%CI: 0.1834–3.173; P = 0.7096; Fig 4A and S3 Table). The patients who had the C allele showed a prolonged OST while those with the T allele showed significantly shorter OST (HR: 0.1165; 95%CI: 0.01773–0.7657; P = 0.0252; Fig 4B and S3 Table). However, promoter methylation and genotypes collectively are not correlated with OST on glioblastoma patients with a hypo-methylated *MGMT* promoter (S2 Fig).

### *MGMT* promoter methylation and genotypes are not predictors for better prognosis in the TMZ-treated glioblastoma patients

**Fig 5:**
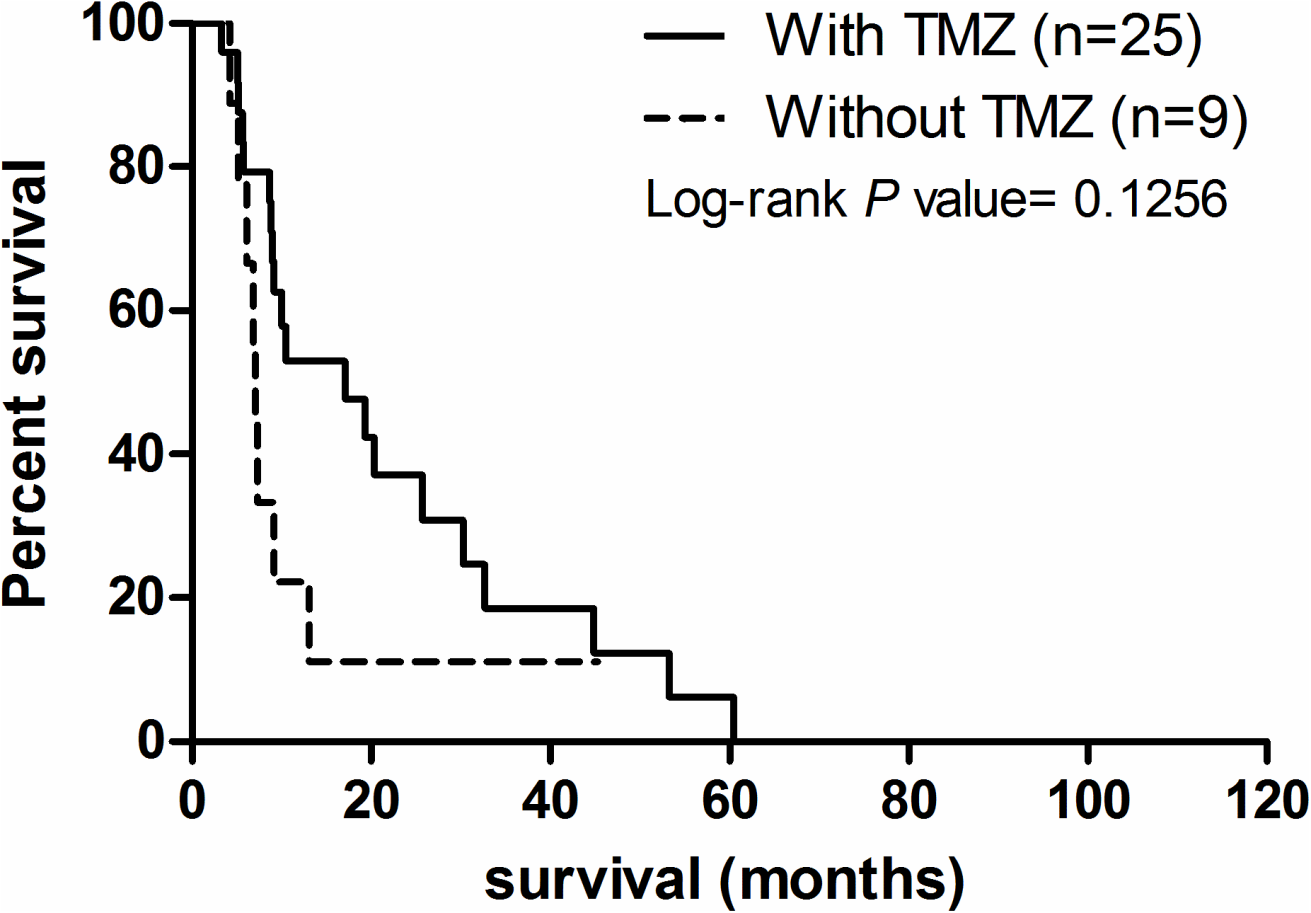
Kaplan-Meier survival analysis on the glioblastoma patients with and without TMZ treatment. Among 34 glioblastoma patients, only 9 patients were not treated with TMZ. The patients treated with TMZ showed a prolonged OST compared to the group without TMZ, although this is not statistically significant (P = 0.1256).

Previous reports suggested that the glioblastoma patients benefited from TMZ therapy and demonstrated a prolonged OST. In our cohort, 25 out of 34 (73.5%) glioblastoma patients were prescribed with TMZ while only five LGG patients were. Of the glioblastoma patients, TMZ-treated patients showed a marginally prolonged OST (HR: 0.4604; 95%CI: 0.1707–1.242; P = 0.1256; Fig 5 and S3 Table). The median survival of the TMZ-treated glioblastoma patients and non-TMZ treated patients differed significantly and are 17.1 months (n = 25) and 7.1 months (n = 9), respectively. Further analysis suggested that rs16906252 genotype is not correlated with OST in TMZ-treated glioblastoma patients (S3A Fig). The patients with hyper-methylated *MGMT* promoter showed a marginally prolonged OST (P = 0.1064, S3B Fig), and the mean OST of the patients with hyper- and hypo-methylated *MGMT* promoter were 25.7 months and 10 months, respectively. On the other hand, the *MGMT* promoter genotypes and methylation were not associated with prolonged OSTs in all glioma patients without TMZ treatment (S4 and S5B Figs), except the LGGs patients having CC genotypes (S5A Fig).

## Discussion

We reported the clinical impact of both promoter methylation and genotypes in *MGMT*, a negative regulator of TMZ treatment, to glioma patients [19]. The patients in this study were all Han-Chinese recruited from a single medical center between 2005 to 2015 to ensure consistent quality of clinical management and patient care. Furthermore, all patients were treated and cared for by the same neurosurgeon. The drawback of this strategy is the number of analyzed patients; the findings should be validated in a larger cohort. Furthermore, analysis of isocitrate dehydrogenase 1 and 2 (*IDH1* and *IDH2*) and 1p19q shall provide further information on patient stratification.

TMZ is a commonly used alkylating agent transferring a methyl group to the O6-guanine, which causes mispairing with thymine during DNA replication and results in apoptosis and cell death. MGMT removes the methyl group resulting in reduction of the therapeutic effect of TMZ [20]. The *MGMT* promoter hyper-methylation has been repeatedly reported as a predictor to effective TMZ treatment in whites and in Han-Chinese, even though some contradictory results were also reported [21–24]. In our study, only marginal association is seen.

Previous studies suggested that the rs16906252 T allele results in reduced promoter activity and MGMT expression [7] and is associated with *MGMT* methylation in glioblastoma [15, 16], colorectal cancer [18, 25, 26], and in a subset of malignant pleural mesothelioma patients[25]. However, the association is not replicated in our Han-Chinese cohort of glioblastomas nor in LGG patients. Since SNP often varies among different ethnic groups, statistical differences might be attributable to ethnic variation. From ExAC and 1000 genome results [17], the minor allele T is 6.857% and 2% in whites respectively, while it is not present in the East Asian population. Our results show that the germline MAF (minor allele frequency) is 0.091 in the Han-Chinese compared to that of 0.1 and 0.14 in the germline genome of the glioma patients of Western European descent [15, 16]. The MAF in brain cancer patients is only slightly different.

The MAF of rs16906252 in tumors went up to 0.153, while 11 tumors underwent C>T somatic mutations. The increase of the rs16906252 T allele might drive a potentially evolutionary advantage, which presents a poor prognosis in our LGGs result. However, the cellular influence of the rs16906252 T allele in LGGs remains to be investigated.

Previous studies showed that glioblastoma patients carrying the T allele with hyper-methylated *MGMT* promoter had significantly better prognosis [16]. The T allele is not correlated with a prolonged OST as reported for glioblastoma patients and for the TMZ-treated glioblastoma patients [23]. In contrast, the LGGs patients showed a surprisingly prolonged OST when carrying the rs16906252 C allele while the T allele is in fact associated with a significantly poor prognosis. While the somatic mutation prevalence in LGGs is higher [27], one explanation is that active MGMT in LGGs might repair DNA damage and further protect the tumor cells from apoptosis and cell death. This facilitates the LGGs tumors becoming malignant.

## Conclusions

In summary, we showed that glioblastoma patients benefited by receiving TMZ treatment and confirmed that the glioblastoma patients with hyper-methylated promoter showed a prolonged OST although the difference is not statistical significant. Although unable to replicate some of the previous findings, we present here some novel findings. We identified a positive correlation between prolonged OST and the rs16906252 CC genotype in LGGs and that the rs16906252 genotypes predict two distinctive outcomes in the hypo-methylated LGG patients. Due to the small number of samples, further validation on a larger cohort of Han-Chinese and east Asians will shed light on more robust conclusions of the clinical utility or *MGMT* genetic testing for brain cancers.

## Supporting information

S1 Fig rs16906252 genotyping by *Hha*1 restriction enzyme digestion and electrophoresis. Genomic DNA was treated with *Hha*1 and electrophoresed in a 3% agarose gel. PCR products (74bp) harboring the rs16906252 T allele prevent *Hha*1 digestion, while that of the C allele is fragmented into 48- and 26-bp fragments. The results of patient’s genotype of the homozygous C, heterozygous CT, homozygous T is illustrated here. U: Undigested; D: Digested.
(TIFF)

S2 Fig Kaplan-Meier survival analysis on the *MGMT* genotype and methylation on the glioblastoma patients. The glioblastoma patients were grouped by their methylation and genotype of the *MGMT* promoter. None of the patient groups showed significantly prolonged OST (P = 0.7358).
(TIFF)

S3 Fig OST of 25 TMZ-treated glioblastoma patients. (A) The CC or CT genotypes did not differentiate patients for prolonged OST (P = 0.5319). (B) The patients with the hyper-methylated *MGMT* promoter showed better survival outcome (median survival time are 17.1 months versus 7.1 months) even though statistically insignificant. (P = 0.1064).
(TIFF)

S4 Fig OST of nine non-TMZ treated GBM patients. (A) These patients had either homozygous C (n = 6) or heterozygous CT genotype (n = 3). However, all showed very short OST except one patient with CC was still alive at the time of analysis. The genotypes do not differentiate OST (P = 0.3668). (B) The promoter methylation does not differentiate OST, except one patient with hypo-methylation showed a prolonged OST (P = 0.3198).
(TIFF)

S5 Fig OST of 33 non-TMZ treated LGG patients. Most LGG patients didn’t receive TMZ treatment except five patients. The result of the genotype (A, P = 0.0435) and the promoter methylation (B, P = 0.9745) on 33 non-TMZ treated patients is similar to that of all LGGs patients.
(TIFF)

S1 Table Patient clinical information. (XLSX)

S2 Table Statistical analysis of the genotypes and the promoter methylation status of the glioma patients. (XLSX)

S3 Table Log-rank test of OST in glioblastoma and the LGG patients. (XLSX)
