# Supplementary figures and images for: The rs16906252:C>T SNP is not associated with increased overall survival or temozolomide response in a Han-Chinese glioma cohort
Source: PLoS One. 2017 Jun 2;12(6):e0178842. doi: 10.1371/journal.pone.0178842 (PMC5456392; doi:10.1371/journal.pone.0178842)

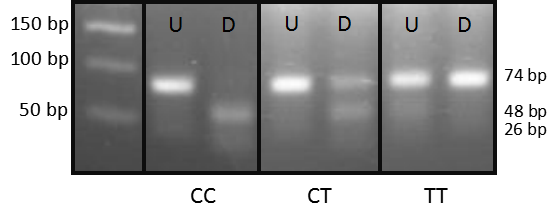

Supplement: S1 Fig — Genomic DNA was treated with Hha1 and electrophoresed in a 3% agarose gel. PCR products (74bp) harboring the rs16906252 T allele prevent Hha1 digestion, while that of the C allele is fragmented into 48- and 26-bp fragments. The results of patient’s genotype of the homozygous C, heterozygous CT, homozygous T is illustrated here. U: Undigested; D: Digested. (TIFF) [file pone.0178842.s001.tiff]

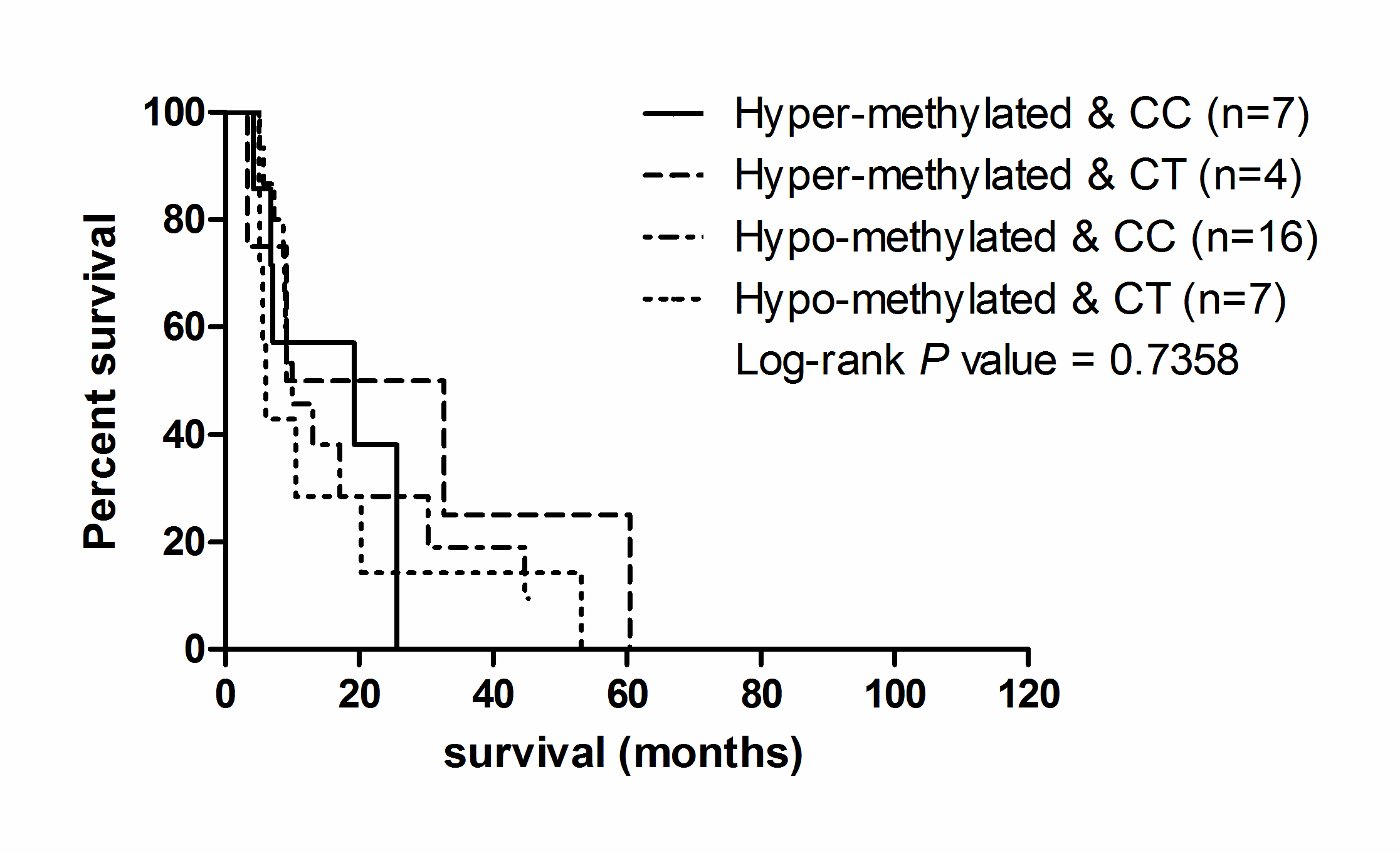

Supplement: S2 Fig — The glioblastoma patients were grouped by their methylation and genotype of the MGMT promoter. None of the patient groups showed significantly prolonged OST (P = 0.7358). (TIFF) [file pone.0178842.s002.tiff]

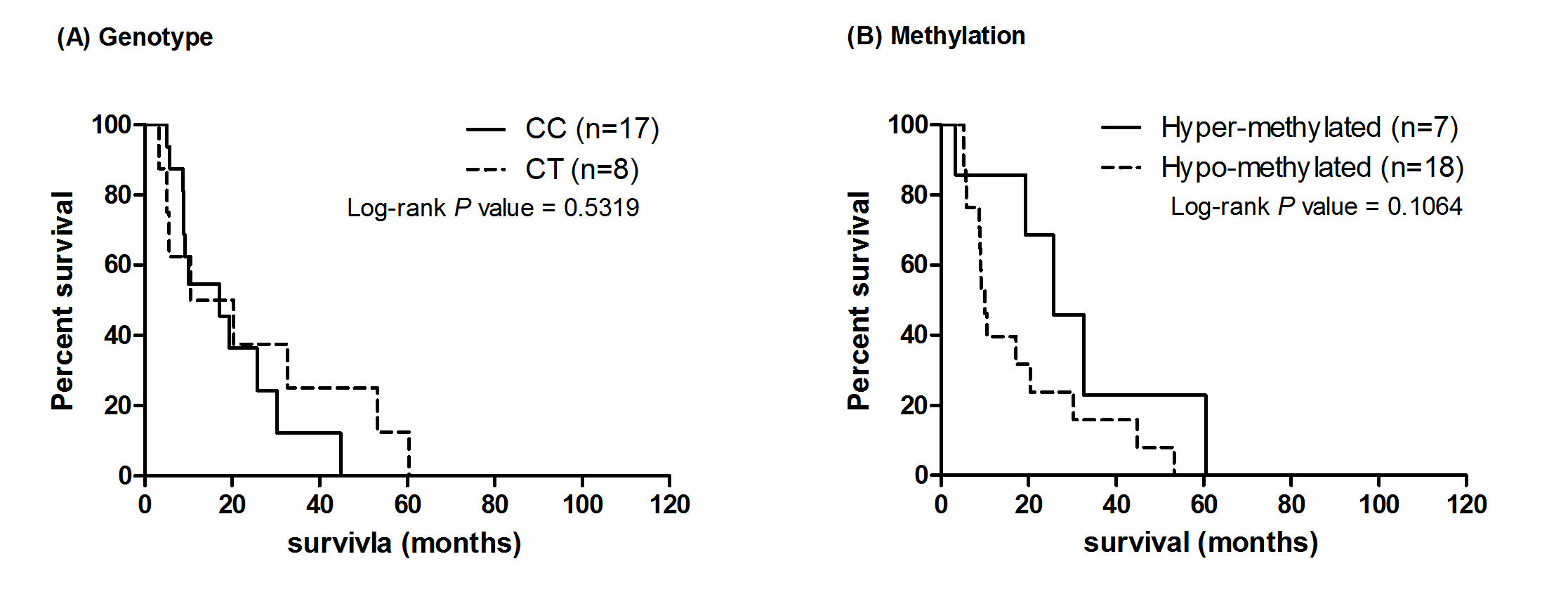

Supplement: S3 Fig — (A) The CC or CT genotypes did not differentiate patients for prolonged OST (P = 0.5319). (B) The patients with the hyper-methylated MGMT promoter showed better survival outcome (median survival time are 17.1 months versus 7.1 months) even though statistically insignificant. (P = 0.1064). (TIFF) [file pone.0178842.s003.tiff]

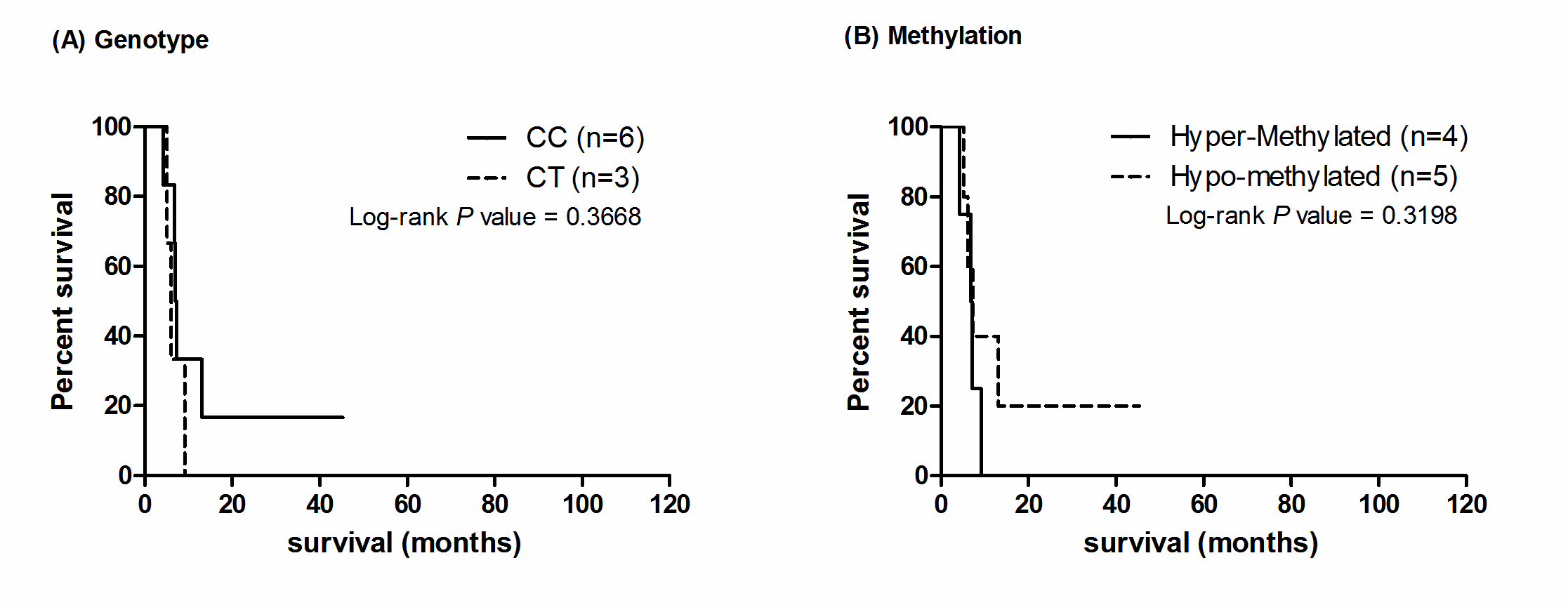

Supplement: S4 Fig — (A) These patients had either homozygous C (n = 6) or heterozygous CT genotype (n = 3). However, all showed very short OST except one patient with CC was still alive at the time of analysis. The genotypes do not differentiate OST (P = 0.3668). (B) The promoter methylation does not differentiate OST, except one patient with hypo-methylation showed a prolonged OST (P = 0.3198). (TIFF) [file pone.0178842.s004.tiff]

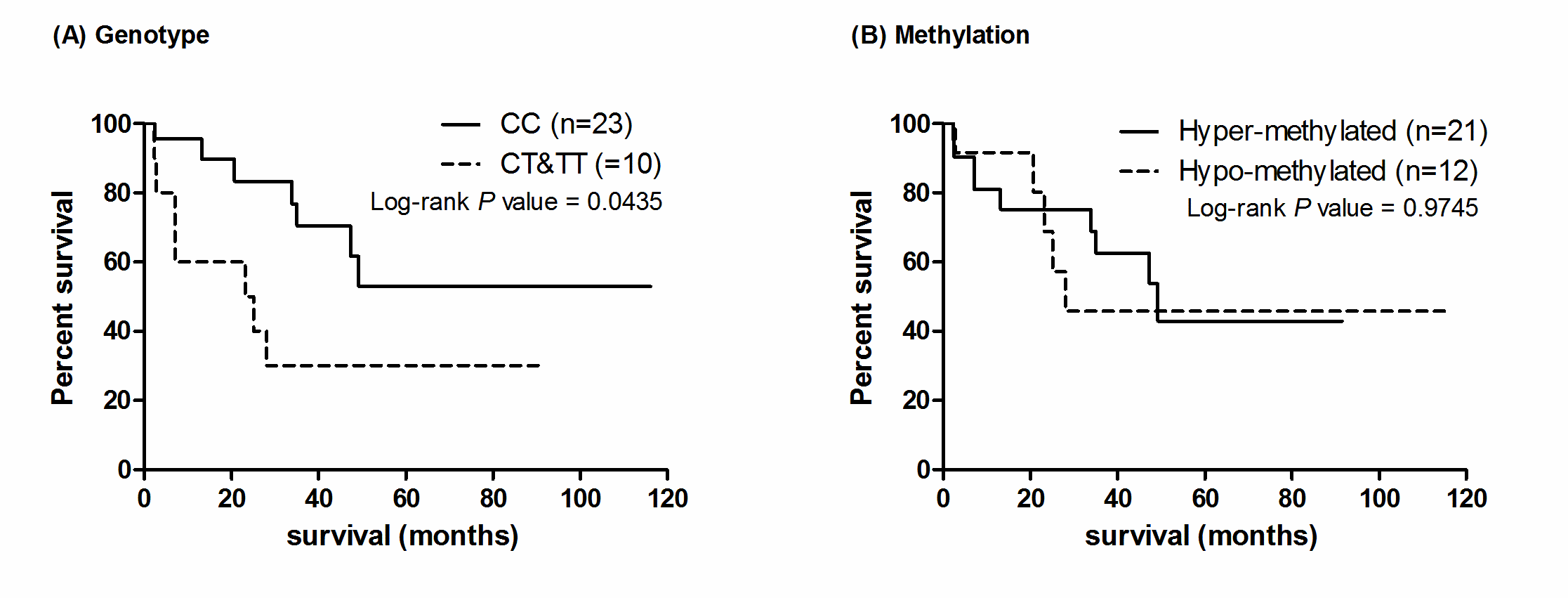

Supplement: S5 Fig — Most LGG patients didn’t receive TMZ treatment except five patients. The result of the genotype (A, P = 0.0435) and the promoter methylation (B, P = 0.9745) on 33 non-TMZ treated patients is similar to that of all LGGs patients. (TIFF) [file pone.0178842.s005.tiff]
